# Advisory Committee on Immunization Practices Recommended Immunization Schedule for Children and Adolescents Aged 18 Years or Younger — United States, 2019

**DOI:** 10.15585/mmwr.mm6805a4

**Published:** 2019-02-08

**Authors:** Candice L. Robinson, Henry Bernstein, José R. Romero, Peter Szilagyi

**Affiliations:** ^1^Immunization Services Division, National Center for Immunization and Respiratory Diseases, CDC; ^2^Zucker School of Medicine at Hofstra/Northwell and Cohen Children’s Medical Center, New Hyde Park, New York; ^3^University of Arkansas for Medical Sciences, Little Rock, Arkansas; ^4^Arkansas Children's Hospital, Little Rock, Arkansas; ^5^Department of Pediatrics, University of California Los Angeles, Los Angeles, California.

At its October 2018 meeting, the Advisory Committee on Immunization Practices (ACIP)* voted to recommend approval of the Recommended Immunization Schedule for Children and Adolescents Aged 18 Years or Younger, United States, 2019. The 2019 child and adolescent immunization schedule summarizes ACIP recommendations, including several changes from the 2018 immunization schedule,^†^ on the cover page, three tables, and notes found on the CDC immunization schedule website (https://www.cdc.gov/vaccines/schedules/index.html). This immunization schedule is recommended by ACIP and approved by the CDC Director, the American Academy of Pediatrics, the American Academy of Family Physicians, and the American College of Obstetricians and Gynecologists. Health care providers are advised to use the tables and the notes together.

ACIP’s recommendations on use of each vaccine are developed after in-depth reviews of vaccine-related data, including disease epidemiology and burden, vaccine efficacy and effectiveness, vaccine safety, quality of evidence, feasibility of program implementation, and economic analyses of immunization policy (*1*). The child and adolescent immunization schedule is published annually to consolidate and summarize updates to ACIP recommendations on vaccination of children and adolescents and to assist health care providers in implementing current ACIP recommendations. The use of trade names of vaccines in this report and in the child and adolescent immunization schedule is for identification purposes only and does not imply endorsement by ACIP or CDC.

For further guidance on the use of each vaccine, including contraindications and precautions, health care providers are referred to the respective ACIP vaccine recommendations at https://www.cdc.gov/vaccines/hcp/acip-recs/index.html. Changes in recommended use of vaccines can occur between annual updates to the child and adolescent immunization schedule. These changes, if made, are available at https://www.cdc.gov/vaccines/acip/recommendations.html).^§^Printable versions of the 2019 child and adolescent immunization schedule and ordering instructions are available on the immunization schedule website.

## Vaccine Changes in the 2019 Immunization Schedule for Children and Adolescents

Vaccine changes in the 2019 immunization schedule for children and adolescents aged ≤18 years include new or revised ACIP recommendations for hepatitis A vaccine (HepA) (*2*), hepatitis B vaccine (Hep B) (*3*), influenza vaccine (*4*), and tetanus toxoid, reduced diphtheria toxoid, and acellular pertussis vaccine (Tdap) (*5*), as well as clarification of the recommendations for inactivated poliovirus vaccines (IPV).

## Changes Affecting Multiple Portions of the Schedule

The overall appearance of the 2019 child and adolescent schedule has been updated because of recommendations resulting from a recent evaluation of the child and adolescent immunization schedule. An internet survey of 249 pediatricians and family medicine physicians was conducted to assess their familiarity with the schedule, the environment in which the schedule is used, the frequency and circumstances of use, and their impressions and preferences on redesigned drafts of the child and adolescent immunization schedule. These changes have been applied to all portions of the immunization schedule, including the cover page, routine immunization schedule (Table 1), catch-up schedule (Table 2), medical indications for each vaccine (Table 3), and notes with details for each vaccine.

**Cover Page.** Changes to the cover page are as follows:

- Guidance on how to use the schedule was added to the top of the document.
- Live attenuated influenza vaccine (LAIV) was added to the vaccine table.
- A Helpful Information section, which includes links to the ACIP recommendations, the General Best Practice Guidelines for Immunization, and the Manual for the Surveillance of Vaccine-Preventable Diseases, has been added.

**Table 1.** Changes to Table 1 (previously known as Figure 1) are as follows:

- A separate row has been added for LAIV.
- A purple bar has been added to the Hepatitis A (HepA) row at age 6–11 months to represent use in infant travelers.
- Within the Tetanus, diphtheria, & acellular pertussis (Tdap: ≥7 yrs) row, the bar for persons aged 13–18 years has been split into a half green and half purple bar to represent catch-up vaccination and use in pregnant adolescents, respectively.

**Table 2.** Changes to Table 2 (previously known as Figure 2) are as follows:

- Minor changes to the order in which guidance is presented in the *Haemophilius influenzae* type b and Pneumococcal conjugate rows were made. The criteria under which no further doses are needed are now presented first, followed by recommendation for those for whom additional doses are indicated.

**Table 3.** Changes to Table 3 (previously known as Figure 3) are as follows:

- A new pink color has been added to the legend, which represents “Delay vaccination until after pregnancy if vaccine indicated.” This color is used in the pregnancy column for human papillomavirus vaccine.
- The Contraindicated and Precaution for vaccination boxes in the legend have been defined with narrative text.
- A row for LAIV has been added.
- The Pregnancy cell in the meningococcal B vaccine row has been changed to the orange Precaution for vaccination color.

**Notes.** The notes (previously known as footnotes) are presented in alphabetical order rather than linked by numerical superscripts as in previous years. Edits have been made throughout the Notes section to harmonize language between the child and adolescent schedule and the adult immunization schedule, where possible. In addition, the following content changes were made:

- The HepA note was revised to include information regarding the use of combined HepA-HepB vaccine in persons aged ≥18 years. A section for international travel has been added with recommendations for vaccination of travelers aged 6–11 months and unvaccinated travelers aged ≥12 months. Homelessness also has been added as an indication for HepA vaccination.
- The HepB note was revised to include information regarding the use of CpG-adjuvanted HepB vaccine and combination HepA-HepB vaccine in persons aged ≥18 years.
- Within the IPV note, a bullet has been added regarding the use of combination vaccines that contain IPV.
- The Influenza vaccines note has been updated to indicate that LAIV can be used during the 2018–19 influenza season. A Special Situations section has been added with information regarding vaccination of persons with a history of egg allergy and circumstances under which LAIV use is not recommended.
- During mumps and meningococcal disease outbreaks, the Additional Information section at the beginning of the notes directs providers to their state or local health department for information regarding vaccination during an outbreak. Therefore, language regarding the use of measles, mumps, and rubella (MMR) vaccine in the setting of a mumps outbreak or the use of meningococcal (groups A, C, W-135, and Y) conjugate (MenACWY) and meningococcal group B (MenB) vaccines in the setting of meningococcal disease outbreaks has been removed from the MMR and meningococcal vaccine notes.
- The Tdap note has been updated to indicate that those persons who received a dose of Tdap or diphtheria and tetanus toxoids and acellular pertussis vaccine (DTaP) at age 7–10 years inadvertently or as part of the catch-up series should still receive the routine dose of Tdap at age 11–12 years. A link to information regarding use of Tdap/tetanus and diphtheria toxoids (Td) for wound prophylaxis also has been added.

## Footnotes

* Recommendations for routine use of vaccines in children and adolescents are developed by ACIP, a federal advisory committee chartered to provide expert external advice and guidance to the CDC Director on use of vaccines and related agents for the control of vaccine-preventable diseases in the civilian population of the United States. Recommendations for routine use of vaccines in children and adolescents are harmonized to the greatest extent possible with recommendations made by the American Academy of Pediatrics, the American Academy of Family Physicians, and the American College of Obstetricians and Gynecologists. ACIP recommendations approved by the CDC Director become agency guidelines on the date published in the *Morbidity and Mortality Weekly Report*. Additional information about ACIP is available at https://www.cdc.gov/vaccines/acip.

† Past immunization schedules are available at https://www.cdc.gov/vaccines/schedules/past.html.

§ CDC encourages organizations to use syndication as a more reliable method for displaying the most current and accurate immunization schedules on an organization's website rather than copying these schedules to their websites. Use of content syndication requires a one-time step that ensures an organization’s website displays current schedules as soon as they are published or revised; instructions for the syndication code are available on CDC’s website (https://www.cdc.gov/vaccines/schedules/syndicate.html). CDC also offers technical assistance for implementing this form of content syndication (e-mail request to ncirdwebteam@cdc.gov). Changes in ACIP recommendations in the child and adolescent immunization schedule before the next scheduled annual update, if any, are available at https://www.cdc.gov/vaccines/schedules/hcp/child-adolescent.html.
